# Sodium-Glucose Cotransporter-2 (SGLT-2) Attenuates Serum Uric Acid (SUA) Level in Patients with Type 2 Diabetes

**DOI:** 10.1155/2021/9973862

**Published:** 2021-06-17

**Authors:** Mazhar Hussain, Asim Elahi, Abid Hussain, Javed Iqbal, Lubna Akhtar, Abdul Majid

**Affiliations:** ^1^Department of Pharmacology & Therapeutics Sheikh Zayed Medical College/Hospital, Rahim Yar Khan, Punjab, Pakistan; ^2^Department of Medicine CHI Saint Joseph Health Hospital, London, Kentucky, USA; ^3^Department of Nephrology Sheikh Zayed Medical College/Hospital, Rahim Yar Khan, Pakistan; ^4^Department of Medicine Sheikh Zayed Medical College/Hospital, Rahim Yar Khan, Pakistan; ^5^Department of Cardiology, Sheikh Zayed Medical College & Hospital Rahim Yar Khan, Pakistan

## Abstract

**Background:**

Hyperuricemia has a strong association with diabetes mellitus. Hyperuricemia can lead to cardiovascular and renal complications in patients with diabetes. The goal of this study was to compare the effect of sodium-glucose cotransporter-2 (SGLT-2) inhibitors dapagliflozin and empagliflozin on serum uric acid (SUA) levels in patients with type 2 diabetes against traditional oral antihyperglycemic drugs (OADs).

**Methods:**

In this double-blind randomized controlled trial, 70 patients with type 2 diabetes and elevated SUA levels were assigned to two treatment groups. Patients in group A received SGLT-2 inhibitors tablet dapagliflozin 5 mg to 10 mg and empagliflozin 10 mg to 25 mg. Group B patients received OADs such as glimepiride, metformin, sitagliptin, gliclazide, and glibenclamide as monotherapy or combination therapy. The changes in SUA level were primary end points while changes in body weight and body mass index (BMI) from baseline to end point were secondary end points.

**Results:**

After four weeks of treatment, we noted a significant reduction of mean SUA levels in the SGLT-2 inhibitor group from 7.5 ± 2.5 to 6.3 ± 0.8 mg/dl versus comparator group from 7.1 ± 1.8 to 6.8 ± 2.2 mg/dl (*p* = 0.001). Mean body weight was significantly reduced in the SGLT-2 group from 82 ± 10.4 to 78 ± 12.5 kg versus comparator group from 78 ± 13.2 to 79.2 ± 9.7 kg (*p* = 0.001). Similarly, the mean BMI of patients in the SGLT-2 group was significantly reduced from 25.7 ± 3.2 to 24.2 ± 3.2 kg/m^2^ versus comparator group from 27.5 ± 4.2 to 28 ± 3.6 kg/m^2^ (*p* = 0.002).

**Conclusion:**

SGLT-2 inhibitors have a strong potential to decrease SUA levels in patients with type 2 diabetes.

## 1. Introduction

The prevalence of diabetes is growing worldwide and continues to represent a global health challenge. In 2015, the estimated prevalence of diabetes was 8.8%, but projections estimate this will rise to 10.4% by 2040 [1].The International Diabetes Federation estimates that the number of patients with diabetes will reach 552 million in 2030, adding significant morbidity and mortality for patients and social and economic burdens on health systems. Identifying and preventing risk factors for diabetes are urgent to stop the diabetes epidemic [2].

Hyperuricemia is a disorder of purine metabolism. Serum uric acid (SUA) levels are usually elevated in patients with diabetes compared to healthy individuals. Hyperuricemia increases the risk of diabetes in people who have impaired glucose tolerance, and hyperuricemia is highly prevalent in patients with diabetes [3, 4]. SUA is a strong independent, novel risk factor for diabetes [5]. Increases in body weight, waist circumference, dyslipidemia, sedentary lifestyle, hypertension, and insulin resistance are predisposing factors for hyperuricemia in patients with diabetes [6, 7].

Hyperuricemia has a strong association with type 2 diabetes and is a strong predictor for developing metabolic syndrome, diabetes mellitus, and hypertension. Hyperuricemia leads to significant morbidity and mortality in the form of kidney and cardiovascular diseases. Reducing SUA in patients with type 2 diabetes may reduce micro and macro complications [8–10].

Sodium-glucose cotransporter-2 (SGLT-2) inhibitors comprise a novel class of oral antihyperglycemic drugs (OADs) with insulin-independent effects. The unique mechanism of SGLT-2 inhibitors is beneficial to patients with diabetes by inhibiting glucose reabsorption from the proximal tubule of the kidney. SGLT-2 inhibitors have shown promising effects on body weight, blood pressure, dyslipidemia, and inflammation. Given their beneficial effects on the cardiovascular system and good safety profile, SGLT-2 inhibitors are now widely prescribed worldwide [11, 12].

Uricosuric and synthesis inhibitors are the two main drug groups used for the treatment of hyperuricemia. Although uricosuric drugs such as probenecid and losartan act through different urate transporters (e.g., URAT1) than SGLT-2 inhibitors (which affect urate transporter SLC2A9 isoform b), the net effect is the same: both drugs cause an increase in the excretion of uric acid in urine [13]. Some studies have shown that SGLT-2 inhibitors have a strong potential to reduce SUA levels.

Therefore, the present study was conducted to determine SGLT-2 inhibitor's effect on SUA levels after four weeks of treatment in patients with type 2 diabetes.

## 2. Materials and Methods

We conducted this four-week double-blind randomized controlled trial at a government clinic and five private clinics in the District of Rahim Yar Khan, South Punjab, Pakistan, from February to March 2020. Three hundred eighty overweight patients with type 2 diabetes on OAD agents were screened for elevated SUA levels. Of this group, 70 patients were enrolled in the study according to our inclusion and exclusion criteria. To be included in the study, patients must have a body mass index (BMI) < 28 kg/m^2^, SUA levels (>7.0 mg/dl for males and >6.0 mg/dl for females) with adequate glycemic control (defined as glycated hemoglobin [HbA1c] <7%), and blood pressure within the reference range (<140/90 mmHg). Patients with a history of smoking, alcohol use, and cardiovascular and renal disease were excluded from the study. Also, a complete history was taken to screen for drugs that can affect uric acid levels, such as thiazide diuretics, aspirin, niacin, statins, anticancer agents, immunosuppressants, and pyrazinamide. Patients taking any antigout drug were also excluded from the study. Patients were also asked to avoid all fructose-containing beverages and foods for the duration of the study.

Patients were randomly assigned to one of two groups by simple random number generation via computer software. Patients in group A (*n* = 35) were treated with daily empagliflozin tablet 10 mg to 25 mg and dapagliflozin 5 mg to 10 mg, and patients in group B (*n* = 35) continue their routine OADs either alone or in combination as the standard treatment of diabetes. These OADs were glimepiride, metformin, sitagliptin, gliclazide, and glibenclamide. Dosages of the drugs were adjusted according to patient fasting blood glucose and random blood glucose level testing. Patients, investigators, and laboratory staff were blinded to the study plan throughout clinical trial.

The changes in SUA level were primary end points while changes in body weight and body mass index (BMI) from baseline to end point were secondary end points.

All clinical and laboratory parameters were measured by following standard guidelines and methods. The study design was approved by the institutional review board of Sheikh Zayed Medical College/Hospital Rahim Yar Khan ( 26/IRB/SZMC/SZH). All study participants provided written informed consent.

## 3. Data Analysis

Values were expressed as mean ± standard deviation and percentages. Numeric data were analyzed using SPSS Statistics for Windows, Version 18.0. (Chicago: SPSS Inc.). A *t*-test was used to compare the changes between the test groups. A paired *t*-test was applied to compare each group's changes, while the Mann-Whitney *U*-test and *t*-test were applied to compare changes between groups from baseline to four weeks. A *p* value <0.05 was considered statistically significant.

## 4. Results

All patients completed the study with none lost to follow-up. In group A, 20 received empagliflozin while 15 patients received dapagliflozin. In group B, 35 patients received their routine OADs. The safety and tolerability profile of empagliflozin and dapagliflozin were quite good, and no adverse effects were recorded during the study.

The baseline demographic characteristics of study participants are presented in Table 1. SUA levels were higher in both groups at the start of the study. Serum uric acid levels significantly decreased in the SGLT-2 inhibitor group from 7.5 ± 2.5 mg/dl to 6.3 ± 0.8 mg/dl, while the control group's SUA levels went from 7.1 ± 1.8 mg/dl at baseline to 6.8 ± 2.2 mg/dl by the end of the study period (*p* = 0.001; Table 2).

Mean body weight was significantly reduced in the SGLT-2 group from 82 ± 10.4 kg to 78 ± 12.5 kg compared to the comparator group, which had a mean body weight of 78 ± 13.2 kg at baseline and 79.2 ± 9.7 kg at the end of the study period (*p* = 0.001). Similarly, BMI was reduced significantly from 25.7 ± 3.2 kg/m^2^ to 24.2 ± 3.2 kg/m^2^ in the SGLT-2 inhibitor group while the control group's mean BMI at baseline was 27.5 ± 4.2 kg/m^2^ and 28 ± 3.6 kg/m^2^ by the end of the study (*p* = 0.002).

## 5. Discussion

To the best of our knowledge, this represents the first study conducted in Pakistan to determine the four-week effect of SGLT-2 inhibitors (dapagliflozin and empagliflozin) on SUA in patients with type 2 diabetes. Both drugs significantly reduced SUA as compared to control with good safety and tolerability profile.

SGLT-2 inhibitors are widely prescribed for patients with diabetes as monotherapy and combination therapy. Their mechanism of action is unique among conventional OADs. They have an insulin-independent effect and increase the excretion of glucose in the urine. These drugs also have beneficial effects on conventional risk factors of cardiovascular disease [14, 15].

SGLT-2 inhibitors also lower SUA levels in various clinical studies [11, 13]. The only US Food and Drug Administration-approved SGLT-2 inhibitors are canagliflozin, dapagliflozin, empagliflozin, and ertugliflozin. Among those, dapagliflozin and empagliflozin are available in Pakistan. A study by Hao et al. [16] concluded that dapagliflozin significantly reduced SUA levels in patients with uncontrolled diabetes who were hospitalized over 24 hours. We noted similar results, but participants in this study have adequate glycemic control, and our study duration was four weeks.

Our results align with a meta-analysis that showed dapagliflozin significantly reduced SUA by 1.14 mg/dl at a dose of 10 mg daily. However, in our study, both dapagliflozin and empagliflozin reduced uric acid by 1.21 mg/dl at different doses. We did not analyze the dose-dependent effects of the SGLT-2 inhibitors. Moreover, in these studies, the status regarding drugs affecting SUA was lacking. Our study excluded all patients receiving drugs that would affect SUA levels [17, 18].

A meta-analysis of 62 studies by Zhao et al. [18] concluded that empagliflozin reduced SUA more significantly than dapagliflozin. However, dapagliflozin showed dose-dependent uric acid-lowering effects. In our study, both drugs significantly reduced SUA levels. Also, our study did not compare dapagliflozin against empagliflozin in changes to SUA level. Similarly, a systematic review and meta-analysis of 12 randomized trials over a follow-up period of 28 ± 22 weeks on empagliflozin (10 mg or 25 mg) revealed that it significantly reduced blood pressure, body weight, HbA1c, and SUA [19].

A meta-analysis of 31 studies on 13,650 diabetic patients demonstrated that SGLT-2 inhibitors significantly decreased SUA levels compared with placebo, canagliflozin 0.62 mg/dl, dapagliflozin 0.64 mg/dl, and empagliflozin 0.71 mg/dl [20]. Our study showed a greater reduction of SUA by 1.21 mg/dl, likely because we excluded all risk factors and drugs that can affect SUA levels.

The proposed mechanisms by which SGLT-2 inhibitors decrease SUA include the expression of glucose transporter 9 isoform 2 in the kidney tubules that causes the excretion of D-glucose and uric acid in urine [21]. Other studies postulated that SGLT-2 inhibitor causes dose-dependent excretion of glucose in the urine, leading to more exchange of uric acid in the apical membrane of tubular cells. This causes more release of uric acid from the blood and reduces uric acid concentration in serum [22, 23, 24]. A recent study showed that empagliflozin reduced SUA levels in mice by upregulation of the ATP binding cassette subfamily G member 2 (junior blood group) via the 5′-adenosine monophosphate- (AMP-) activated protein kinase (AMPK)/protein kinase B/cAMP-response element-binding protein signaling pathway [25]. Moreover, reducing the risk factors of hyperuricemia such as body weight, blood pressure, and insulin resistance by SGLT-2 inhibitors has additional beneficial effects in patients with diabetes.

Our study's main strength was the exclusion of risk factors that can affect SUA levels, such as obesity, hypertension, chronic kidney disease, and uncontrolled diabetes. We also excluded patients taking any drugs that can affect SUA levels. However, our study was limited in that we did not check urine for a complete examination for uric acid, and we did not compare empagliflozin and dapagliflozin for effects on SUA levels.

## 6. Conclusions

SGLT-2 inhibitors have a strong potential to decrease SUA levels in patients with type 2 diabetes. SGLT-2 inhibitors can be administered in patients with diabetes with abnormal SUA levels. Hyperuricemia is one of the potential risk factors for cardiovascular and kidney diseases. Therefore, lowering SUA levels is crucial to prevent morbidity and mortality in patients with type 2 diabetes.

Further studies with larger sample sizes and longer durations should be conducted to illuminate the uric acid-lowering effects of SGLT-2 inhibitors.

## Figures and Tables

**Table 1:** Baseline demographics characteristics (*N* = 70).

Baseline parameters	Group A^a^	Group B^b^	*p* value
Age in years (mean ± SD)	46 ± 11.5	43 ± 13.2	0.629
Male, *n* (%)	24 (68.5%)	20 (57%)	0.170
Female, *n* (%)	11 (31.1%)	15 (42.8%)	0.23
Duration of diabetes in years (mean ± SD)	8.2 ± 4.8	9.2 ± 6.2	0.236
Body weight in kg (mean ± SD)	82 ± 10.4	78 ± 13.2	0.324
BMI in kg/m^2^ (mean ± SD)	25.7 ± 3.2	27.5 ± 4.2	0.763
Blood pressure systolic in mmHg (mean ± SD)	115 ± 10.8	125 ± 8.5	0.671
Blood pressure diastolic mmHg (mean ± SD)	80 ± 4.8	88 ± 3.2	0.162
Fasting blood glucose in mg/dl (mean ± SD)	99 ± 12.7	105 ± 10	0.68
HbA1c (mean ± SD)	6.5 ± 3.2	7.0 ± 2.8	0.682
Serum uric acid level in mg/dl (mean ± SD)	7.5 ± 2.5	7.1 ± 1.8	0.36

^a^SGLT-2 inhibitors (*n* = 35): empagliflozin (*n* = 20) and dapagliflozin (*n* = 15). ^b^Standard OAD therapy (*n* = 35). *t*-test between two groups. Abbreviations: BMI: body mass index; HbA1c: glycated hemoglobin; OAD: oral antihyperglycemic drug; SD: standard deviation; SGLT-2: sodium-glucose cotransporter-2.

**Table 2:** Comparison of uric acid changes at baseline and after treatment.

Parameters	Group A^a^		*p* value^b^	Group B^c^		*p* value^b^	*p* value^d^
	Baseline	End point		Baseline	End point		
Body weight in kg (mean ± SD)	82 ± 10.4	78 ± 12.5	0.005	78 ± 13.2	79.2 ± 9.7	0.02	0.001
BMI in kg/m^2^ (mean ± SD)	25.7 ± 3.2	24.2 ± 3.2	0.001	27.5 ± 4.2	28 ± 3.6	0.056	0.002
Fasting blood glucose in mg/dl (mean ± SD)	99 ± 12.7	90 ± 10.2	0.42	105 ± 10	95.5 ± 10	0.042	0.62
Serum uric acid level in mg/dl (mean ± SD)	7.5 ± 2.5	6.3 ± 0.8	0.001	7.1 ± 1.8	6.8 ± 2.2	0.042	0.001

^a^SGLT-2 inhibitors (*n* = 35): empagliflozin (*n* = 20) and dapagliflozin (*n* = 15). ^b^Comparison within groups. ^c^Standard OAD therapy (*n* = 35). ^d^Comparison of changes of each variable between the two groups. Abbreviations: BMI: body mass index; HbA1c: glycated hemoglobin; OAD: oral antihyperglycemic drug; SD: standard deviation; SGLT-2: sodium-glucose cotransporter-2.

## Data Availability

The data used to support the findings of this study are available from the corresponding author upon request.
